# 3D Simulations of Intracerebral Hemorrhage Detection Using Broadband Microwave Technology

**DOI:** 10.3390/s19163482

**Published:** 2019-08-09

**Authors:** Andreas Fhager, Stefan Candefjord, Mikael Elam, Mikael Persson

**Affiliations:** 1 Department of Electrical Engineering, Chalmers University of Technology, 412 96 Gothenburg, Sweden; 2 MedTech West, Sahlgrenska University Hospital, 413 45 Gothenburg, Sweden; 3 Inst of Neuroscience and Physiology, Dept. of Clinical Neurophysiology, Sahlgrenska Academy, Göteborg University and with Neuro-Division, Sahlgrenska University Hospital, 413 45 Gothenburg, Sweden

**Keywords:** intracranial hemorrhage, stroke, machine learning, subspace classifier, microwave technology, FDTD modeling

## Abstract

Early, preferably prehospital, detection of intracranial bleeding after trauma or stroke would dramatically improve the acute care of these large patient groups. In this paper, we use simulated microwave transmission data to investigate the performance of a machine learning classification algorithm based on subspace distances for the detection of intracranial bleeding. A computational model, consisting of realistic human head models of patients with bleeding, as well as healthy subjects, was inserted in an antenna array model. The Finite-Difference Time-Domain (FDTD) method was then used to generate simulated transmission coefficients between all possible combinations of antenna pairs. These transmission data were used both to train and evaluate the performance of the classification algorithm and to investigate its ability to distinguish patients with versus without intracranial bleeding. We studied how classification results were affected by the number of healthy subjects and patients used to train the algorithm, and in particular, we were interested in investigating how many samples were needed in the training dataset to obtain classification results better than chance. Our results indicated that at least 200 subjects, i.e., 100 each of the healthy subjects and bleeding patients, were needed to obtain classification results consistently better than chance (*p* < 0.05 using Student’s *t*-test). The results also showed that classification results improved with the number of subjects in the training data. With a sample size that approached 1000 subjects, classifications results characterized as area under the receiver operating curve (AUC) approached 1.0, indicating very high sensitivity and specificity.

## 1. Introduction

Microwave technology is currently researched for use in stroke and trauma diagnostics. The technique has the potential to change dramatically how, when, what, and where care can be delivered to these patients [1]. The aim is to facilitate early diagnosis of these life-threatening conditions, preferably already before arrival to the hospital, thereby improving medical outcome. In both stroke and trauma, the single most important factor for saving lives and for successful patient recovery is the time from incidence to treatment. Current state-of-the art diagnosis is normally performed at hospitals, which often causes significant delays of adequate treatment. Therefore, there is a need for diagnostic methods that are suitable for prehospital use. At the forefront of microwave diagnostics research are groups in Sweden [2,3], Austria [4,5,6,7] and Australia [8,9,10], who have reached a stage of pre-clinical and clinical trials on stroke and trauma patients. Significant efforts, including experimental lab test on phantoms, were published in [11]. The review by Fhager et al. [1] provided an overview of the historical development and state-of-the-art of microwave diagnostics for the brain. In addition, several research groups are active in the field of the development of systems and algorithms, and much of their progress in the area was summarized in the book [12].

Research in microwave diagnostic applications for brain has sprung from microwave-based breast imaging for cancer detection. In the field of breast diagnostics, a major goal is quantitative or qualitative imaging of the dielectric parameters, even if classification based on machine learning has been suggested. A review of classification methods, primarily of time-domain data, can be found in [13], and a more recent recent analysis of machine learning-based breast tumor classification can be found in [14]. Imaging techniques are in general computationally demanding due to their need for detailed full wave numerical modeling of the antenna array and imaging domain. The iterative reconstruction algorithms require multiple simulations of the microwave propagation in the antenna system to refine the reconstructed images. It is not uncommon to have reconstruction times of several hours for 3D imaging, even on powerful parallel computers, and this is clearly not a feasible solution that can be accommodated on portable equipment [1]. Telemedicine solutions, which could be used to transfer data to a computer center, have been proposed for prehospital applications [15]. Radar-based algorithms, also explored in breast cancer imaging, are much more computationally efficient, but only capable of reconstructing relative images of scatterers [10,16].

For the microwave technique to be applicable for prehospital use, a real-time imaging method is needed. As a method to enable extremely short reconstruction times, differential imaging has been proposed in monitoring situations [17]. We proposed a similar technique exploiting differential imaging, where anatomical MRI or CT images are segmented into tissue classes and thereafter assigned tissue-specific dielectric values to obtain dielectric maps of the patient’s head, and used these maps as a priori starting guesses in the image reconstruction algorithm [18]. A bleeding, which manifests itself as a local variation in the dielectric distribution of the normal head, can thus be effectively reconstructed with linear methods by only imaging the corresponding deviation from the healthy brain map. This potentially leads to very fast reconstruction and could be particularly useful in monitoring applications, where data such as an MRI scan of the patient is already available. However, much research remains before such a method can be used in practice. Development of fast forward solvers that can be integrated into reconstruction algorithms could potentially also enable imaging of the brain [19,20]. Reconstruction times on the order of seconds for 2D and about 20 min for 3D reconstructions are within reach in a not so distant future.

We have also shown, in three different clinical studies, that classification based on a machine learning method may be used for stroke and trauma diagnostics [2,3]. This method can generate real-time results even on devices with low computational power, such as tablets. However, it is necessary to first collect clinical data from patients and healthy volunteers needed to train the algorithm. Clinical studies are both time-consuming and costly, and the number of included patients should be kept at a minimum. On the other hand, machine learning methods usually require large amounts of training data to reach high accuracy [21,22]. It is therefore necessary to study how the amount of training data relates to the performance and accuracy of the algorithm used in our earlier work.

In two of our previous studies, we used a 2D simulation model employing the Finite Element Method (FEM) to study the potential to detect, localize [23], and estimate the size [24] of traumatic intracranial bleeding. In [23], a total of 1000 bleeding subjects were modeled, and a high classification accuracy of 94–100% was reached. However, that study aimed at localizing the bleeding, and no evaluation of accuracy as a function of the number of samples was performed. In [24], the classification accuracy as a function of the number of samples was investigated. It was concluded that to detect bleeding with high accuracy, a sample size in the order of 100 patients with bleeding and 100 healthy subjects was needed, whereas accurate estimation of size required larger sample sizes. Both studies were modeling subdural hematoma in 2D, a crescent-shaped intracranial bleeding situated between the skull bone and the brain. Subdural hematoma is different from intracerebral hemorrhage, which is the most common type of bleeding for stroke patients. Intracerebral bleeding is situated within the brain tissue and is commonly spherically shaped and generally smaller in volume than subdural hematoma. A 2D model however represents a significant simplification of a real head and the scattering that occurs in a real measurement scenario, and consequently, it is unclear to what extent results from such a model can be generalized to real measurements made in 3D. The development of a 3D model is therefore important, and it allows for more realistic representations of the variability between patients and healthy persons. This would also constitute an important step in the theoretical assessment of the classification technique. The ultimate goal of the 3D modeling that is initiated in this paper is to create a realistic simulation environment from which it is possible to predict experimental and clinical results accurately. Such a model must naturally be made in 3D in order to resemble a real-world experimental setup. Running thousands of highly-resolved 3D FDTD is feasible, but still a computationally-expensive and time-consuming task. Relating 3D results back to the results obtained in 2D is therefore interesting in order to investigate to what degree results obtained in 2D are comparable.

In this paper, we present a numerical simulation study based on realistic 3D Finite-Difference Time-Domain (FDTD) modeling of the skull, brain, and modeling of spherical intracerebral bleeding to investigate the theoretical performance of the classifier used in our previous work [2,3,24], as a function of the number of subjects used for training. A simplified antenna array consisting of dipoles was modeled. One single normal head model was used as the starting point, which in every simulation was randomly rescaled to resemble a natural variation of skull sizes. The bleeding was randomly placed inside the brain with randomly-varying volumes of realistic sizes. To improve the realism of mimicking typical microwave systems, noise was added before the simulation data were fed into the classifier. Finally, the classifier performance was evaluated using a nested cross-validation scheme.

With the results presented in this and previously-published studies [23,24], a general understanding of the performance of the classification-based detection scheme is sought. The aim is to get input to further development of microwave diagnostics of intracranial bleeding and for planning of future clinical trials.

## 2. Method

This section contains a description of the methods used in the electromagnetic simulations to generate scattering data and how head models, with and without bleeding, were generated. It also describes the classification method and the cross-validation scheme used to evaluate the performance of the classifier.

### 2.1. Electromagnetic Modeling and Simulations

In this study, healthy head models, i.e., without bleeding, were created together with patient head models with bleeding. The purpose was to investigate the performance of the classification method as a function of the number of training subjects. A large dataset was therefore generated with a total of 1000 subjects, where 500 were patients and 500 healthy models. While the number of patients could be increased even further to study the theoretical limits of the classification scheme, this study was limited to 1000 subjects. There were three reasons for this. First, empirical experience has shown that clinically-relevant performance can be reached with less than 1000 patients. Second, since future training data will be generated from clinical studies, it is not realistic to conduct studies with several thousand patients considering cost, complexity, and time. Third, the computation time and memory consumption increase drastically with the number of samples, and modeling and classifying more than 1000 patients was impractical.

**Figure 1:**
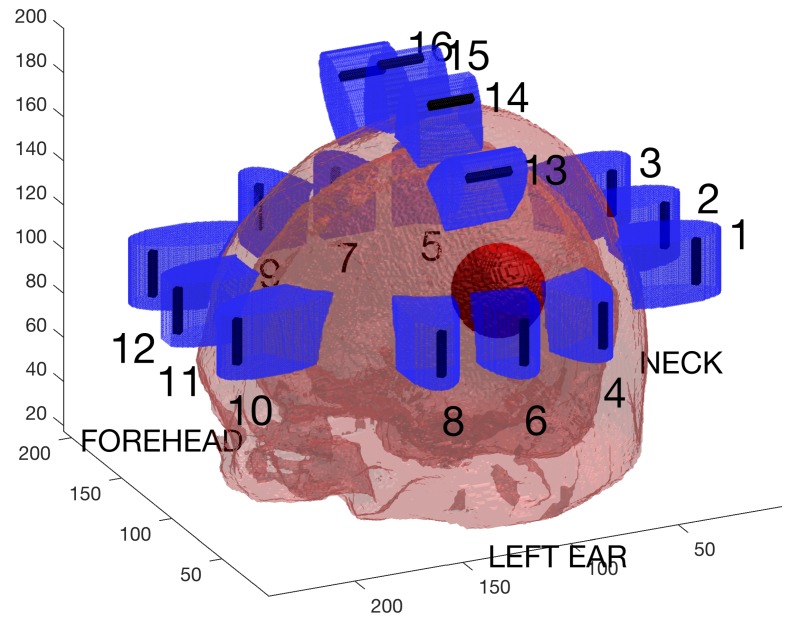
Sixteen antennas were placed around the head model used in the simulations. The figure also shows one example of a spherical bleeding model in dark red color and the containers with deionized water in blue.

An antenna array consisting of 16 dipole antennas of length 21 mm, was placed around the head. Twelve antennas were vertically aligned and placed with their center points in a plane surrounding the head model. Four of the antennas were aligned horizontally and placed above the head. The positioning of antennas was chosen to resemble the configurations used in our earlier experimental and clinical work, but the number of antennas was larger. Similarly, the antenna length was chosen to produce a resonance frequency in a range corresponding to systems used in clinical studies [2,3], slightly below 1 GHz. Like the patch antennas used in the measurements, dipole antennas radiate in all directions, and thus, the multi-path effects in the simulation model should be present in both these simulations and earlier measurements. The antenna array and head model, including an example of a spherical bleeding, are illustrated in Figure 1. To ensure good coupling of signals into the skull, each antenna was placed in containers of deionized water in contact with the skull. The water containers were modeled as cylinders with elliptic cross-sections extending 5 mm above and below the endpoints of the antennas. These containers are shown in blue color in Figure 1.

The computational model was derived from an anatomical tissue model of a healthy human head from BrainWeb [25]. The original model contained tissue types: background, Cerebrospinal Fluid (CSF), grey matter, white matter, fat, muscle, skin, and skull. It also included glial matter and connective tissue, which however were not included in our model. The reason is that we are not aware of any publication of their dielectric properties. The total volumes of these tissues are relatively small, about 0.08% and 4%, respectively, of the total head model volume. Their structures are similar to fat and gray matter, respectively. Hence, we used the dielectric properties of fat to model glial matter and those of gray matter to model connective tissue. In addition, a layer of hair was added on the surface of the head. Again, we are not aware of any measurements of the dielectric properties of hair. Hair is primarily composed of the protein alpha-keratin, which is also the most common component of nails. Data of dielectric properties of nail-tissue were available and were used for modeling hair. In this model, we neglected the possibility of air being present between individual hairs. In theory, air in between the hairs could be modeled with a dielectric mixture law. However, in the clinical measurements [2,3], the antennas were applied with a certain pressure, thereby compressing the hair and removing the air. This is a reasonable procedure if the patient’s hair has a normal or small thickness; for patients with thick hair, the only solution might be shaving.

The dielectric properties of tissue were obtained from [26], which is a large study of dielectric tissue properties and often used as a basis for numerical modeling. In this paper, the Cole–Cole model parameters describing the dispersive behavior of different tissues were determined from experimental data. Unfortunately, it was not possible to use Cole–Cole models directly in our FDTD code [27]. Instead, Debye models were fitted to the data according to Equation (1).

(1) $$\epsilon \left(\omega \right)=\epsilon_{\infty }+\frac{\epsilon_{\mathrm{static}}-\epsilon_{\infty }}{1+j\omega \tau }+\frac{\sigma }{j\omega }.$$

**Figure 2:**
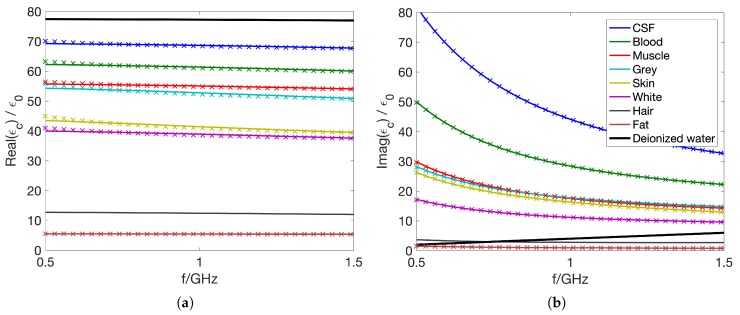
This figure shows the real (**a**) and imaginary (**b**) part of the complex permittivity of the tissues as a function of frequency. Solid lines represent the fitted Debye models, and cross-shaped markers show sampled values of the original Cole–Cole data [26,28].

Here, ϵ(ω) is the complex permittivity as a function of angular frequency, ω. The permittivities at the high and low frequency ends are denoted $\epsilon_{\infty }$ and $\epsilon_{\mathrm{static}}$, respectively; τ is the relaxation time; and σ denotes the static conductivity. In a least squares optimization scheme over the frequency range 0.5–1.5 GHz, the Debye parameters $\epsilon_{\mathrm{static}}$, $\epsilon_{\infty }$, σ, and τ were determined for each tissue. The dielectric properties of the tissues, as well as blood used in this simulation study are shown in Figure 2, together with the properties of deionized water, modeled with $\epsilon_{\mathrm{static}}=77.5\epsilon_{0}$, $\epsilon_{\infty }=4.65\epsilon_{0}$, σ=0.0 S/m, and τ=8.8 ps, with $\epsilon_{0}$ denoting the vacuum permittivity. Solid lines represent the fitted Debye models, and cross-shaped markers show sampled values of the original Cole–Cole data [26,28]; both real and imaginary parts of the complex permittivity are shown in Figure 2a,b, respectively. As an approximation of hair, the dielectric properties for nails published in [28] were used.

**Figure 3:**
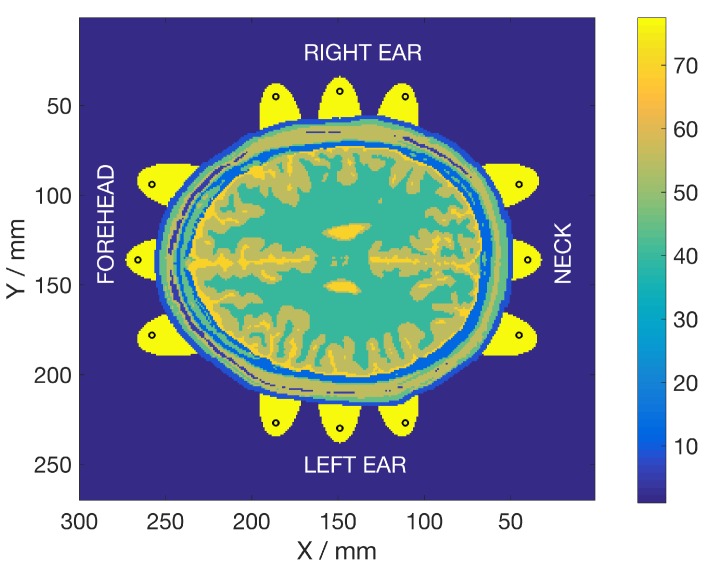
Cross-section of the head at the center of the vertical antennas. The figure shows the relative static permittivity, $\epsilon_{\mathrm{static}}$, in the Debye model. Antenna positions are marked with black circles and the immersion medium as elliptically-shaped cylinders. The color bar shows relative permittivity.

As an example, a 2D cross-sectional image of the static permittivity, i.e., the corresponding permittivity at the static point in the Debye model, calculated as $\epsilon_{\mathrm{static}}$, of a healthy subject is shown in Figure 3.

To simulate patients with intracranial bleeding caused by stroke or trauma, a spherical volume of blood was inserted into the modeled head. In reality, bleeding forms a volume that infiltrates and/or redistributes the normal tissue. For simplicity, such complex structural deformations were ignored in this study, and instead, the bleeding was realized by replacing the original brain tissue dielectric parameters with the dielectric data of blood in the spherical bleeding volume.

**Figure 4:**
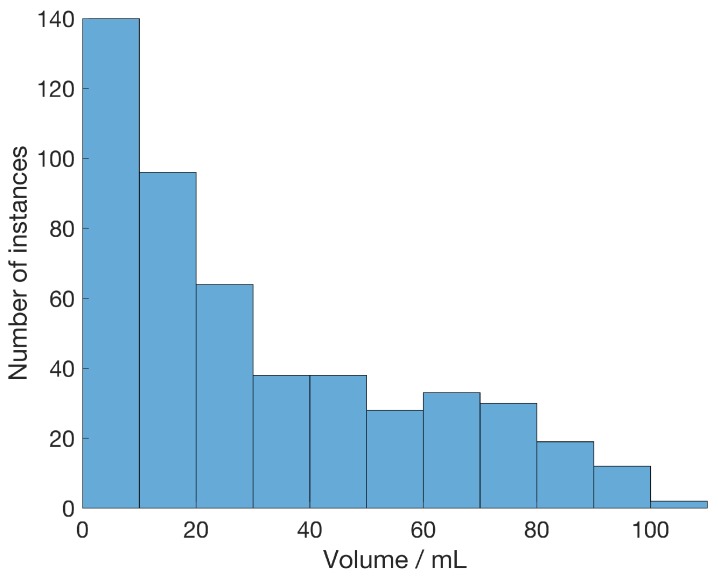
In the simulations, 1000 different head models were used, where 500 with bleeding were included, all within the range 0–105 mL. The histogram shows the number of bleeding instances of each volume represented in the dataset with bleeding subjects.

The position and volume of the spherical bleeding were randomly selected such that the spherical volume fitted entirely within the cranial cavity. Realistic bleeding volumes in the range,  0–105 mL were used, and the distribution of volumes in the simulation models representing the 500 subjects with bleeding are shown in Figure 4. The procedure for placing the bleeding in the model was as follows. 1. A random radius of the sphere was drawn. 2. The potential center points inside the brain tissue were calculated such that the entire sphere fit within the cranial cavity. 3. A center point was randomly drawn from the potential center points. 4. The bleeding was added to the head model (this was done before the rescaling of the model according to the description below).

The original head model from BrainWeb [29] was contained in a matrix with 181×217×181 elements, and each voxel had dimensions 1×1×1 mm${}^{3}$. This model was used as the origin for the 1000 models, but to introduce a more realistic individual variation, it was randomly rescaled in the x-, y-, and z-directions before each simulation. The rescaling was done such that the variation in sizes of the model followed the same distribution found among humans. Head size data, as well as data for several other body parts, within the American population were collected in [30]. Size data for both genders were available, and it was reported that male heads are on average larger than female heads, 10 mm larger measuring from ear to ear, 16 mm larger forehead to neck, and 3 mm larger top of the head to a horizontal line just below the eye ball. For our study, the mean value of male and female average head sizes was used. A new distribution was obtained by adding the two distributions for men and women, leading to a distribution with the first percentile of women and the 99th percentile of men, to model a normal distribution of head sizes for both genders. In Table 1, the published head size data from [30] is reprinted together with the combined head size data used in our study, labeled “computational model”.

One note is that body sizes can vary between populations in the world, but also with generations. The original model from BrainWeb had the distances between ears of 180 mm, forehead to neck of 210 mm, and top of the head to the bottom of the eye ball of 150 mm. For each simulation, the head model was randomly rescaled to have 1st, 50th, and 99th percentiles according to the data in the column “computational model” in Table 1. The healthy head models and the head models used for the bleeding patients were independently generated. The procedure for rescaling was as follows: 1. Three independent random numbers were drawn from a normal distribution defined according to the data shown in the “computational model” columns. 2. Scaling factors for the x, y, and z coordinates were calculated (the ratios of the new desired measures and original measures). 3. The original head model matrix was resampled using nearest neighbor interpolation (the resampling was implemented with the MATLAB command imresize). 4. The rescaled head model was inserted into the FDTD simulation matrix. Thereafter, the hair was added, and its thickness was randomly set to an integer in the range 0–4 mm.

The FDTD method was used to calculate transmission coefficients, represented in terms of amplitude A(ω) and phase ϕ(ω) as complex numbers S(ω)=A(ω)exp[iϕ(ω)]. Transmission coefficients were computed between all combinations of antennas in the frequency range 0.4–1.2 GHz with a frequency step of approximately 3 MHz. Random white noise was added to the simulated data, and the noise was modeled to resemble, as closely as possible, the noise seen in measurements with Vector Network Analyzers (VNA), i.e., as amplitude and phase noise. Noise was therefore added to the amplitude and phase of the transmission coefficients as:

(2) $$S_{noisy}\left(\omega \right)=\left[A\left(\omega \right)+A_{n}\left(\omega \right)\right]\exp[i\left(\phi \left(\omega \right)+\phi_{n}\left(\omega \right)\right)].$$

The noise was generated as $A_{n}\left(\omega \right)=\sigma N$ and phase $\phi_{n}\left(\omega \right)=\arctan\frac{\sigma N}{\left|A(\omega )\right|}$. *N* is a random number drawn from a standard normal distribution, and σ is the desired standard deviation of the added noise. The standard deviation was chosen such that $\sigma =10^{\left(n_{f}/20\right)}$, and $n_{f}$ is the noise floor, expressed in dB.

### 2.2. Classification of Modeled Subjects and Assessment of Diagnostic Performance for Different Numbers of Subjects

The classification algorithm used here was based on the same principles as in [2,3]; a detailed description was provided in Persson et al. [2]. The classifier outputs a subspace distance to each class, which is a measure of similarity to the class. Each simulated measurement’s data $x_{c}^{i}$, where *c* is the class label and *i* denotes the sample number, were combined into a vector of complex numbers that were fed into the classifier. The frequency range was set to 0.4–1.2 GHz (267 frequency points), and all 120 transmission coefficients $S_{ij}$ were used, resulting in a vector composed of 267×120=32040 elements. No further preprocessing of the raw simulation data was conducted. In this study, the two classes for the binary classification task were patients with bleeding and healthy subjects without bleeding. All training data samples $x_{c}^{i}$ in one class were joined into a matrix $X_{c}=\left[x_{c}^{1},x_{c}^{2},\ldots ,x_{c}^{t_{c}}\right]$, where $t_{c}$ denotes the number of training samples in class *c*, from which the class subspaces $U_{c}$ were estimated by singular value decomposition [2]. The distances to class “bleeding” and class “healthy’ were computed by projecting the data vector $x_{c}^{i}$ onto the subspaces $U_{bleeding}$ and $U_{healthy}$, and the difference between the distances was used as the decision value for the classifier. A Receiver Operating Characteristic (ROC) curve was derived by adjusting the decision value, i.e., a varying offset was added so that either the healthy or bleeding class was favored. In other words, on one extreme, the bleeding detection sensitivity was maximized at the cost of more misclassifications of healthy subjects, and on the other extreme, the specificity (correct classification of healthy subjects) was maximized at the cost of more misclassifications of bleeding subjects, while in the intermediary range, the two classes were separated into different degrees as represented by the sensitivity and specificity on the ROC. Scatter plots [2,3] showing the decision values for a group of subjects were used to visualize the degree of separation.

The diagnostic performance was assessed by calculating the Area Under the ROC Curve (AUC) [2,3]. An AUC of ∼0.5 corresponds to a useless diagnostic test, no better than chance. An AUC of 1.0 constitutes a perfect test classifying all subjects correctly. To cope with the large number of patients in this numerical study while keeping the computation times for the classifier reasonable, a 10-fold Cross-Validation (CV) procedure was used, instead of the leave-one-out CV procedure used in earlier studies. Ten-fold CV means that the algorithm was trained on approximately 90 % (nine out of the ten folds) of the observations in the dataset and then tested on the remaining 10 % (the left out fold) of the observations, so that data used for training were kept separate from data used for testing. The procedure was iterated ten times, so that all ten folds were tested.

In order to select tuning parameter settings (subspace dimensions) [2] for the classifier, an inner CV loop was used to estimate optimal settings, while the outer CV loop estimated the final diagnostic performance. This procedure is termed nested cross-validation and was described in detail by Statnikov et al. [31]. It was used by Oliveira and colleagues for the development of breast cancer diagnostics with microwave technology [14]. The nested CV procedure accomplishes simultaneous optimal selection of tuning parameters and unbiased (non-overfitted) estimation of diagnostic performance for the final model [31,32,33]. For simplicity, examples of scatter plots and ROC for visualization were derived using traditional 10-fold CV, with tuning parameters chosen to produce typical and representative diagnostic accuracies.

The diagnostic performance as a function of the number of subjects used for training of the classifier were estimated from stratified subsets of data from the 1000 subjects with an equal number of patients and healthy individuals. The subset data sizes ranged from 30–1000; the specific number of subjects were 30, 40, 50, 60, 100, 200, 300, 500, 750, and 1000. With this choice, the results are presented such that trends in the diagnostic performance can be resolved. For each subset data size, a 10-fold nested CV procedure was conducted, as described above. To assess the statistical variation, the whole procedure was repeated ten times for each subset data size, and the mean and standard deviation of the ten iterations were used as end results.

To evaluate whether the classification performance was better than chance (AUC ∼0.5), a one-tailed Student’s *t*-test was performed, after having confirmed that the data followed a normal distribution using the Anderson–Darling test. The null hypothesis was that the median AUC value for each subset data size equaled 0.5, where small *p*-values cast doubt on the null hypothesis and indicated that the classification performance was AUC > 0.5.

The evaluation of classifier performance was conducted using MATLAB (Version R2016b, MathWorks Inc., Natick, MA, USA) on a computer cluster.

## 3. Results

**Figure 5:**
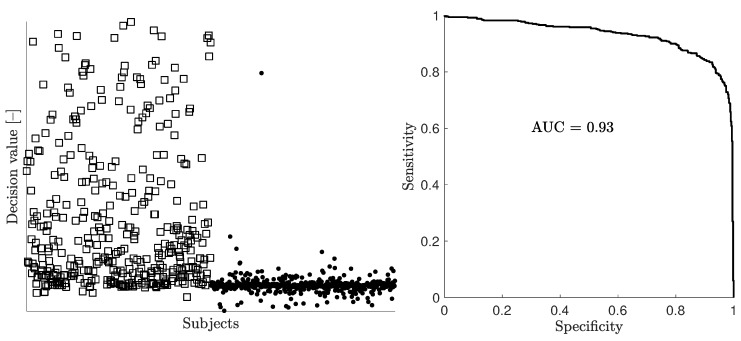
Example of a scatter plot (left) and corresponding ROC/AUC (right) for the full dataset containing 1000 subjects. In the scatter plot, patient models with bleeding (n=500) are shown with squares, whereas healthy subjects (n=500) are shown with dots. For increased clarity, the *y*-axis of the scatter plot has been truncated in order to zoom in on the region where the decision values for the majority of subjects fall, while more extreme values for a minority of subjects are not shown.

**Figure 6:**
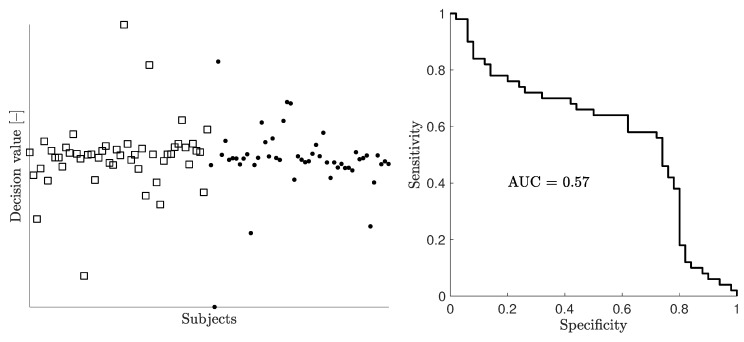
Example of a scatter plot (left) and corresponding ROC/AUC (right) for a random stratified subset containing 100 subjects. In the scatter plot, patient models with bleeding (n=50) are shown with squares, whereas healthy subjects (n=50) are shown with dots.

Figure 5 and Figure 6 show examples of scatter plots and corresponding ROC curves for the full dataset with 1000 subjects and a random stratified subset of 100 subjects, respectively. Half of the subjects were patient models with bleeding, and the other half were healthy models. The scatter plots show the decision value, i.e., the difference between the distance to class “bleeding” and the distance to class “healthy”, for all individual models as evaluated using 10-fold CV. The classifier essentially separated patients from healthy subjects when the full dataset of 1000 subjects was used for the 10-fold CV procedure (Figure 5). For a dataset with 100 subjects, the decision values of both bleeding and non-bleeding subjects were overlapping, and as a consequence, the classifier could not distinguish patients from healthy subjects (Figure 6).

**Figure 7:**
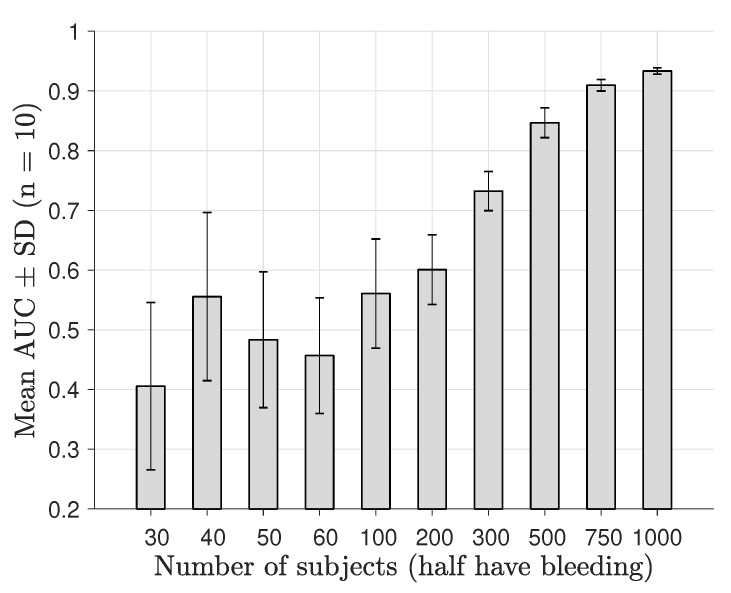
AUC as a function of the number of training data samples, for microwave scattering data with a low amplitude noise floor at −100 dB.

**Figure 8:**
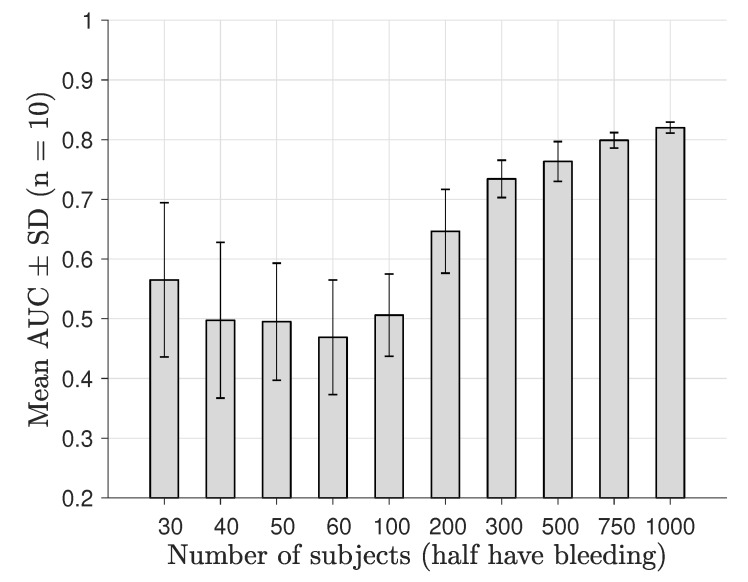
AUC as a function of the number of training data samples, for microwave scattering data with a high amplitude noise floor at −70 dB.

The classification performance as a function of number of subjects derived by nested 10-fold CV is shown in Figure 7 for a noise level of −100 dB, and a corresponding plot for a noise level of −70 dB is shown in Figure 8. We observed that the diagnostic performance rose steadily above AUC ∼0.5 as the subset data size reached 200–300 subjects and that maximum performance was substantially decreased for the −70 dB level. The statistical tests showed that classification performance was higher than AUC∼0.5 for patient sample sizes from 100–200 patients; see Table 2.

## 4. Discussion

In the development of microwave-based diagnostic methods for stroke and trauma diagnostics, early clinical studies with promising results have been reported [2,3]; however, these studies were based on relatively small patient cohorts. While large patient populations are needed to obtain statistically-validated results with narrow confidence intervals for estimated diagnostic performance measures such as sensitivity and specificity, recruitment of large patient cohorts is a challenging task. Theoretical studies, such as the one performed here, are therefore necessary to build knowledge about the underlying principles and further development of the classification algorithm.

The 3D computational model used in this study constitutes a simplified representation of the anatomy and dielectric distribution of a real head and lesions, even if effort has been made to introduce a realistic level of variability between subjects. The model has been used to study the accuracy of a classification algorithm as a function of the number of subjects used for training.

There are also challenges in modeling a fully-realistic lesion. The bleeding model used in this work is a very simplified representation of intracranial bleeding, probably best resembling the situation in the acute phase. Potential secondary effects have not been taken into account. The reason is that the acute phase immediately after the bleeding has occurred is best understood, whereas little is known about the effect on dielectric properties due to secondary effects. In the acute phase, bleeding can be seen as a volume of blood that exerts pressure on the brain and deforms adjacent tissue. A somewhat more complex scenario arises if the blood also mixes with CSF. After the acute phase, the body responds to the injury and lack of oxygenation, e.g., with an inflammatory process and/or increased intracranial pressure. These secondary effects will most certainly influence the dielectric parameters and in some way affect the possibilities of detection. The characteristics of such dielectric changes are however unknown and could not be taken into account in the present study.

It is only recently that machine learning methods have been applied to classification of microwave data, and the understanding of their performance in this realm is limited. The fundamental idea with machine learning-based classification is for the algorithm to learn to recognize the particular features in the data originating from a lesion and to differentiate these features from naturally-occurring variations. An important goal with this study is therefore to study the classification algorithm as applied to microwave data and intracerebral bleeding detection. In general, these types of algorithms require large amounts of data for the learning phase, and a good understanding of the learning phase of the algorithm and how it affects the classification results is important. The results here showed that 200 subjects or more were needed to obtain statistically-significant classification results better than chance (p<0.05). In our earlier 2D study [24] of subdural hematoma, i.e., shallow bleeding in close proximity to the antennas, the same amount of subjects (i.e., 200 subjects) in the training data yielded a detection performance with both sensitivity and specificity larger than 0.9. The reasons for the higher accuracy in the previous study could be two-fold. First, the variability in a 2D geometry was less than in 3D, e.g., the bleeding geometry was more complex in 3D. Second, the bleeding in the 2D study was in general larger and not so deep-seated as here. The finding that classification results improved with an increased training dataset size was however consistent between the two studies.

The validity of our findings are naturally limited to the particular application and techniques used, such as the antenna array and broadband measurement technique, the simulation model and its implementation, and the classification algorithm. Furthermore, currently, we cannot provide a detailed explanation of the theoretical basis for the accuracy improvement as a function of the size of training data. The current study represents a step towards increased understanding of the size of training data needed for microwave diagnostics based on machine learning. We encourage other research groups to investigate other use cases and compare the results to the present study, with the common goal to develop methods that require as small an amount of patients for training as possible.

In this study, normal variations of head sizes were introduced together with randomly-placed bleeding of realistic volumes, as would typically be found in patient populations with intracerebral brain injuries. Small volumes were dominating, as seen in Figure 4. This would make the classification problem more challenging compared to including a greater proportion of large bleeding instances. To somewhat ease the computational burden in this study, dipole antennas were used instead of patch antennas. Furthermore, this study used 16 antennas whereas, earlier clinical works used 8, 10, and 12 antennas. Given all the approximations and simplifications of this model, it is not expected that the results in this study will be directly comparable to our earlier clinical work. Nevertheless, we believe the simplified model used here is of sufficient accuracy to draw conclusions about trends and to build conceptual knowledge of the classification algorithm and its behavior when applied to microwave data.

## 5. Conclusions

In this study, we used a head model from Brain Web and introduced the variability of head sizes to a degree that is representative for the adult population. Further, we included intracranial bleeding of randomly-distributed size and position and used the models to generate simulated scattering data of an antenna array on the head. We investigated how a machine learning-based classification algorithm performs in distinguishing healthy subjects from subjects with bleeding based on the microwave data.

The results showed that at least 200 subjects were needed for the training to obtain statistically-significant classification results better than chance. Even with smaller amounts of data, the results were sometimes indicating a detection capability better than chance, but with large variability. Another important conclusion in this study is that the results improved when including more subjects in the training. With 1000 subjects used for the training, an AUC above 0.9 for data with low noise was reached. The curve has not yet converged, and it looks like even further improvements can be obtained with more subjects.

By adding a level of noise, which was in the range of what could be realistically expected in practice, it was seen that 200 subjects were needed to obtain performance better than chance consistently. Classification accuracy was still improving with larger numbers of subjects, but with a lower AUC of about 0.8 for 1000 subjects.

Further numerical and experimental phantom studies would be valuable to understand for example the effect of the number, position, and orientation of antennas and to understand how the position and volume of the bleeding affects the accuracy of the detection.

One should be careful in direct comparison of these results to earlier clinical results and experiments, as they are specific to the details in the modeling and antenna configuration. Even if detailed comparison to clinical work cannot be made, our opinion is that these results can reveal the trends and overall characteristics of the results. We therefore conclude that large-scale clinical trials with several hundred patients are needed for a practical and clinical verification of the technique.

## Figures and Tables

**Table 1:** Head size data of the American male and female populations (in mm) presented with their 1st, 50th, and 99th percentiles; the data are reprinted from [30]. The head size data used for this study were obtained by adding the two distributions for men and women, and the resulting percentiles are shown in the three columns labeled “computational model”.

	Male			Female			Computational Model		
Percentiles	1	50	99	1	50	99	1	50	99
Ear-Ear	142	155	169	132	145	159	132	150	169
Forehead-Neck	180	196	214	162	180	198	162	188	214
Top-Below Eye	112	125	137	109	122	135	109	123.5	137

**Table 2:** Results of one-tailed Student’s *t*-test. Values in bold are statistically significant (p<0.05).

N	30	40	50	60	100	200	300	500	750	1000
−70 dB	0.073	0.524	0.562	0.834	0.394	<0.0001	<0.0001	<0.0001	<0.0001	<0.0001
−100 dB	0.961	0.135	0.664	0.891	**0.041**	<0.0001	<0.0001	<0.0001	<0.0001	<0.0001
